# Supplementary material for: Predictors of drug survival for biologic and targeted synthetic DMARDs in rheumatoid arthritis: Analysis from the TRA Clinical Electronic Registry
Source: PLoS One. 2021 Apr 30;16(4):e0250877. doi: 10.1371/journal.pone.0250877 (PMC8087098; doi:10.1371/journal.pone.0250877)
Supplement: S2 File — (PDF) [file pone.0250877.s002.pdf]

Demographics
RF and CCP

病患名:

性別:

出生日:

身份證字號:

病歷號:

就診日:

備註一: RA發病日期：

備註二: RF第一次數值：18 / CCP第一次數值：100.62

備註三: 感染

建立日期 2021/03/04

Comorbidity

Smoking

未填寫	Never	Ever	Current

Alcohol

未填寫	有	無	已戒

HLA-B27

未填寫	有	無	未驗

RF 說明

未填寫	有	無	不明

Anti-CCP 說明

未填寫	有	無	不明

DM(糖尿病) 說明

未填寫	有	無	不明

HTN(高血壓) 說明

未填寫	有	無	不明

IGRA 說明

未填寫	有	無	不明

HBV(B型肝炎) 說明

未填寫	有	無	不明

HCV(C型肝炎) 說明

未填寫	有	無	不明

DAS28

觸痛關節總數

8

腫脹關節總數

0

病患疼痛程度(最痛關節)(cm)

好 不好 0

病患整體疾病活動程度(cm)

好 不好 3

醫師 整體疾病活動程度(cm)

好 不好 2

紅血球沉降速率

31 mm/hr

C反應蛋白

22.69 mg/L

Medication

建檔日期	病歷號	用藥資訊(分類 - 藥物名 - 用藥期間)
2020/05/07		NSAIDs - etoricoxib - 2013/09/02~
2014/02/10		boDMARDs - abatacept - 2014/02/10~
