# Supplementary material for: Predictors of drug survival for biologic and targeted synthetic DMARDs in rheumatoid arthritis: Analysis from the TRA Clinical Electronic Registry
Source: PLoS One. 2021 Apr 30;16(4):e0250877. doi: 10.1371/journal.pone.0250877 (PMC8087098; doi:10.1371/journal.pone.0250877)
Supplement: Attachment — Submitted filename: PLOS One.doc [file pone.0250877.s003.doc]

**Dear Author,**

Thanks for submitting your research manuscript entitled “Seropositivity Associated with Drug Survival of Biologic and Targeted Synthetic DMARDs in Rheumatoid Arthritis: Analysis from the TRA Clinical Electronic Registry”

**Major concerns**

***Please find out the following comments***

- The rationale for the study is not mentioned or incomplete regarding Targeted Synthetic DMARDs in Rheumatoid Arthritis.
- The rationale behind the Analysis from the TRA Clinical Electronic Registry is also not clear and should be specified in the introduction and discussed in the results.
- Overall the whole manuscript must be again thoroughly revised to correct grammar, punctuation, and syntax. The authors should use small bites of sentences to tell their views or literature review in a comprehensive way, rather than using complicated and hectic lines and sentences.

**Title:**

The author should have revised the manuscript's title concerning the core concept of the research. More like a statement to the reviewer, the title does not display any clear claim to the study regarding drug Survival of Biologic and Targeted Synthetic DMARDs in Rheumatoid Arthritis.

**Abstract:**

- The rationale behind this research is not well explained, and several major concerns still constrain the reviewer's enthusiasm for publishing this manuscript.
- The abstract has no specific information about the study schedules and creates uncertainty
- The author failed to offer a straightforward conclusion to the abstract and should be more descriptive and follow the research’s rationale.

**Introduction:**

- In addition, the basic literature is not well written and does not even include any literature on alternative approaches in the treatment of Rheumatoid Arthritis.
- What were the studies in existing research that were included? These are the basic questions which the author needs to address.
- The authors should present the justification behind the selection of o three major groups: tumor necrosis factor-alpha (TNF-α) inhibitors, non-TNF-α inhibitors, and tofacitinib.
- In the intro, the author should have furnished the individual references for these.
- The statement and conclusive remarks provided in the introduction are confusing and not matched with the clear observation associated with abstract, results and discussion parts.

**Material and methods:**

- The author should have given the rationale or study schedule behind the research. The author does not mention any specific protocol in a selection of data source and study population.
- What are the altered results related to the combination treatment? The author must provide answers to all these types of questions.
- The M&M writing pattern is not made scientifically, creating confusion through the format of disorganized writing regarding the electronic registry.
- In order to support the assessment of all mentioned parameters in his study, the author should provide all the source documents and data he has followed for all electronic procedures.

**Results:**

- Results need more clarification and significant justification. Differentiating between the outcome and the discussion sections is quite difficult. It looks like; there is no scientific clarity, and follow continuous paragraph writing without any point discussion.
- Authors fail to articulate the outcomes properly in Baseline demographic data, Factors associated with 3-year drug survival, Drug survival curves by biologics-exposure status, Drug survival curves by causes of discontinuation, and Individual drug survival curves by RF and ACPA positivity. Therefore, the findings should be cautiously revised.
- The author should strengthen and organize the result section more significantly and scientifically.
- The author should provide the raw data in scanned PDF or excel format (including institutional certificates of ethical approval, practical record register, practical record entry, or specific instrument/equipment data entry).

**Discussion:**

- Significant scientific statements and observations are without proper reference in the discussion. When explaining the outcomes, the author needs to be very cautious.
- Author's needs to orient and provide the scientific justification to correlate all the parameters he performed, in a very scientific and significant manner.
- In both the discussion and the conclusion, the aims, rationale, and future perspectives are not evident clearly.
- The discussion is usually organized at the beginning to address all the observations and evaluate them at the end. It makes the results easier to contextualize and simpler to comprehend. Furthermore, a minimal critical analysis should be provided.
- According to the reviewer, a more thorough analysis of the findings is needed in the view of the current literature. To address the outcome measures/results separately and how they correlate with the existing literature, it would be better if the author restructured to take a more critical approach.
- The reviewer feels that the conclusion part is somewhere that seems out of focus.
- The reviewer believes that the conclusion section appears to be out of sight somewhere.
